# Blurred lines: Primary metabolic machinery coopted for specialized metabolism in tomato trichomes

**DOI:** 10.1093/plphys/kiac532

**Published:** 2022-12-01

**Authors:** Rachel E Kerwin

**Affiliations:** Department of Biochemistry and Molecular Biology, Michigan State University, East Lansing, Michigan 48824, USA

Plants synthesize an extensive cache of specialized metabolites that mediate environmental interactions and increase their fitness (Pichersky and Gang, 2000). These small molecules are built from primary metabolite precursors and tailored by various enzymes, resulting in structurally diverse compounds. In contrast to the products of primary metabolism, which are largely ubiquitous, individual plant lineages synthesize, but a fraction of the specialized metabolites are found among all plants (Pichersky and Gang, 2000). Additionally, specialized metabolites tend to be synthesized in specific cells or tissues related to their function.

Acylsugars are a group of sticky defense metabolites found in members of the nightshade family (Solanaceae). They are synthesized in trichomes and exuded onto the epidermis, where they protect against herbivory, disease, and desiccation (Leckie et al., 2012; Luu et al., 2017; Feng et al., 2021). Acylsugars are constructed from relatively simple building blocks: sugars and acyl-coenzyme A (CoA) derived from branched-chain amino acids.

Across the Solanaceae family, acylsugar structural variation arises from differences in sugar core identity and acylchain composition (Fan et al., 2019). Notably, acylsugar abundance varies widely as well. For example, acylsugar accumulation in cultivated tomato (*Solanum lycopersicum*) is approximately 1% of dry leaf weight, while wild tomato (*S. pennellii*) accessions accumulate acylsugars up to 20% of dry leaf weight (Fobes et al., 1985).

Core steps of acylsugar biosynthesis, including esterification of acyl-CoA onto the sugar moiety, have been well-characterized in cultivated tomato and other Solanaceae species (Fan et al., 2019). In contrast, upstream pathway steps, such as the formation of acyl-CoA substrates, have not been fully elucidated. Similarly, the genetic basis of differential acylsugar accumulation is not well understood (Leckie et al., 2012).

In this issue of *Plant Physiology*, Ji et al. (2022) took advantage of variation between cultivated and wild tomato to identify enzymes involved in acylsugar biosynthesis. Using LA716, a high acylsugar-producing wild tomato accession, and VF36, a low acylsugar-producing cultivated tomato accession, they generated an F_2_ mapping population with varied acylsugar abundance. Next, they performed trichome metabolite screening on 114 F_2_ individuals and selected 10 high acylsugar producers (HIGH-F_2_s) and 10 low acylsugar producers (LOW-F_2_s) for transcriptome sequencing. From this dataset, the authors identified 331 genes that were significantly differentially expressed between the HIGH-F_2_s and LOW-F_2_s. These 331 differentially expressed genes (DEGs) were overrepresented for functions such as “acyltransferase activity” and “fatty acid metabolic process,” further implicating them in acylsugar metabolism.

To narrow their candidate gene list, the authors compared DEGs from this study with previously identified DEGs between high and low acylsugar-producing wild tomato accessions (Mandal et al., 2020), revealing 73 overlapping genes. The authors also screened for protein-coding sequence differences between cultivated and wild tomato, uncovering hundreds of genes likely under positive selection, including three genes that overlap with the two sets of DEGs and exhibit trichome-enriched expression, an attribute shared by characterized acylsugar metabolic genes (Figure 1A). These three candidate genes, encoding (1) a Rubisco small subunit (*SpRBCS1*; *Sopen07g006810*), (2) a beta-ketoacyl-(acyl-carrier protein) reductase (*SpKAR1*; *Sopen05g009610*), and (3) an induced stolon-tip protein-like member (*SpSTPL*; *Sopen05g032580*), were selected for further analysis to determine their roles in trichome acylsugar metabolism.

**Figure 1:**
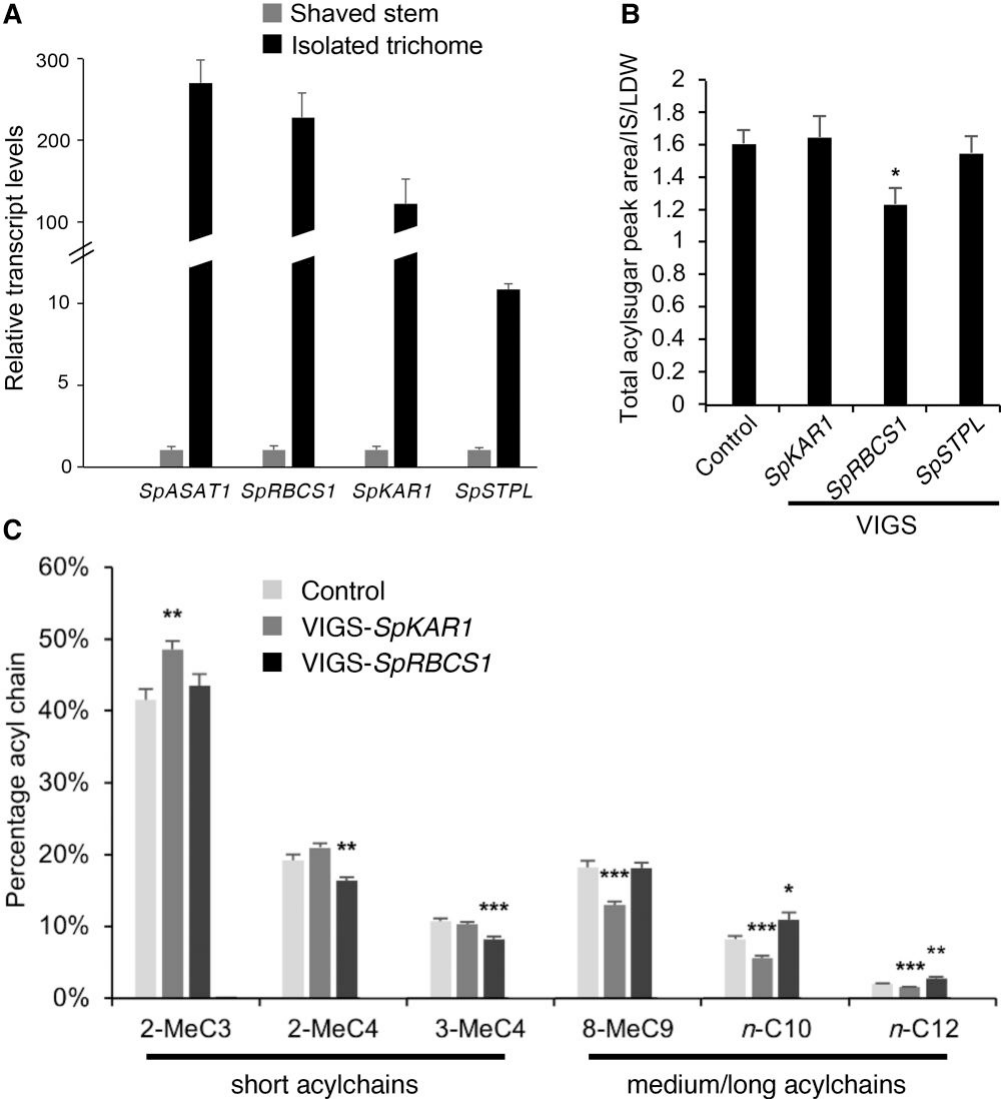
Identification and characterization of candidate genes involved in trichome acylsugar biosynthesis in tomato. A, Transcript abundances of three acylsugar metabolism gene candidates, (1) a Rubisco small subunit (*SpRBCS1*; *Sopen07g006810*), (2) a beta-ketoacyl-(acyl-carrier protein) reductase (*SpKAR1*; *Sopen05g009610*), and (3) an induced stolon-tip protein-like member (*SpSTPL*; *Sopen05g032580*), are strongly enriched in isolated trichomes. Trichome-enriched acylsugar acyltransferase 1 from wild tomato (*Solanum pennellii*) (*SpASAT1; Sopen12g002290*) is included for comparison. Error bars indicate Se (*n* = 5 individuals). B and C, VIGS was employed to test *in planta* gene function in wild tomato. Error bars indicate Se (*n* = 10, 12, 11, and 12 individuals for control, VIGS-*SpKAR1*, VIGS-*SpRBCS1*, and VIGS-*SpSTPL* plants). *P-value < 0.05, **P-value < 0.01, ***P-value < 0.001, ****P-value < 0.0001, Dunnett's post hoc test. B, Total acylsugars decreased in VIGS-silenced *SpRBCS1* plants but not VIGS-silenced *SpKAR1* or *SpSTPL* plants. C, Analysis of acylsugar acylchain composition revealed altered profiles for the VIGS-*SpKAR1* and VIGS-*SpRBCS1* mutants. The VIGS-*SpKAR1* mutant exhibited a decrease in medium/long (8-MeC9, n-C10, and n-C12) acylchains and an increase in short (2-MeC3) acylchains. In contrast, the VIGS-*SpRBCS1* mutant exhibited an increase in medium/long (n-C10 and n-C12) acylchains and a decrease in short (2-MeC4 and 3-MeC4) acylchains. Modified from Ji et al. (2022; Figures 2C, 3A, and 3D). IS, internal standard; LDW, leaf dry weight.

To test *in planta* gene functions, the authors used virus-induced gene silencing (VIGS) in the wild tomato accession LA716 (Figure 1B). VIGS of *SpRBCS1*—but not *SpKAR1* or *SpSTPL*—resulted in lower acylsugar abundance in wild tomato trichomes. Further, silencing either *SpRBCS1* or *SpKAR1* affected acylchain composition, but in different directions. Specifically, the ratio of short to medium/long acylchains decreased when *SpRBCS1* was silenced and increased when *SpKAR1* was silenced (Figure 1C).

In wild tomato trichomes, medium and long acylchains are produced via acyl-CoA elongation, which is mediated by fatty acid synthase—an enzyme complex that includes a KAR1 protein (Slocombe et al., 2008; Mandal et al., 2020). KAR1 is part of a large family of short-chain dehydrogenase/reductase (SDR) proteins often involved in plant specialized metabolism. Enrichment of short acylchains in trichome acylsugars of the VIGS-*SpKAR1* mutant demonstrates that this enzyme is involved in acylchain elongation in wild tomato. Phylogenetic analysis revealed that *SpKAR1* clusters more closely to primary metabolic SDRs from bacteria than plant SDRs involved in specialized metabolism, suggesting that a primary metabolic enzyme was coopted independently for acylsugar biosynthesis.

To better understand the role of *SpRBCS1* in acylsugar biosynthesis, the authors measured carbon isotope levels in trichomes and trichome-less stems, observing higher ^12^C to ^13^C ratios in trichomes. As Rubisco preferentially incorporates ^12^C over ^13^C during photosynthesis, ^12^C enrichment in trichome metabolites reflects greater carbon recycling in this tissue compared with stems. Importantly, this ^12^C enrichment was lower in trichomes of the VIGS-*SpRBCS1* mutant, along with acylsugar abundance. These results support the hypothesis that *SpRBCS1* supplies the trichome acylsugar machinery with recycled carbon.

*SpRBCS1* is one of five *RBCS* genes annotated in *S. pennellii*, the only trichome-enriched copy, and presumably the only copy functioning in specialized, rather than primary, metabolism. Phylogenetic analysis revealed that *SpRBCS1* is part of a trichome-expressed *RBCS* gene cluster restricted to the Solanaceae, suggesting that it was coopted from primary metabolism during acylsugar pathway evolution. Interestingly, trichome-enrichment of *RBCS1* is much less pronounced in cultivated tomato, which may account for the variation in acylsugar accumulation observed between wild and cultivated tomato. Results from this study shed light on acylsugar biosynthesis and underscore how duplication of primary metabolic genes followed by nucleotide divergence and gene expression changes is an important mechanism through which specialized metabolite pathways evolve.

## References

[kiac532-B1] Fan P , Leong BJ, Last RL (2019) Tip of the trichome: evolution of acylsugar metabolic diversity in Solanaceae. Curr Opin Plant Biol 49: 8–16 31009840 10.1016/j.pbi.2019.03.005 PMC6688940

[kiac532-B2] Feng H , Acosta-Gamboa L, Kruse LH, Tracy JD, Chung SH, Nava Fereira AR, Shakir S, Xu H, Sunter G, Gore MA, et al (2021) Acylsugars protect *Nicotiana benthamiana* against insect herbivory and desiccation. Plant Mol Biol 109(4–5): 505–522 34586580 10.1007/s11103-021-01191-3

[kiac532-B3] Fobes JF , Mudd JB, Marsden MPF (1985) Epicuticular lipid accumulation on the leaves of *Lycopersicon pennellii* (Corr.) D’Arcy and *Lycopersicon esculentum* Mill. Plant Physiol 77(3): 567–570 16664099 10.1104/pp.77.3.567 PMC1064565

[kiac532-B4] Ji W , Mandal S, Rezenom YH, McKnight TD (2023) Specialized metabolism by trichome-enriched Rubisco and fatty acid synthase components. Plant Physiol 191(2): 1199–1213 36264116 10.1093/plphys/kiac487 PMC9922422

[kiac532-B5] Leckie BM , De Jong DM, Mutschler MA (2012) Quantitative trait loci increasing acylsugars in tomato breeding lines and their impacts on silverleaf whiteflies. Mol Breed 30(4): 1621–1634

[kiac532-B6] Luu VT , Weinhold A, Ullah C, Dressel S, Schoettner M, Gase K, Gaquerel E, Xu S, Baldwin IT (2017) O-acyl sugars protect a wild tobacco from both native fungal pathogens and a specialist herbivore. Plant Physiol 174(1): 370–386 28275149 10.1104/pp.16.01904 PMC5411141

[kiac532-B7] Mandal S , Ji W, McKnight TD (2020) Candidate gene networks for acylsugar metabolism and plant defense in wild tomato *Solanum pennellii*. Plant Cell 32(1): 81–99 31628166 10.1105/tpc.19.00552 PMC6961621

[kiac532-B8] Pichersky E , Gang DR (2000) Genetics and biochemistry of secondary metabolites in plants: an evolutionary perspective. Trends Plant Sci 5(10): 439–445 11044721 10.1016/s1360-1385(00)01741-6

[kiac532-B9] Slocombe SP , Schauvinhold I, McQuinn RP, Besser K, Welsby NA, Harper A, Aziz N, Li Y, Larson TR, Giovannoni J, et al (2008) Transcriptomic and reverse genetic analyses of branched-chain fatty acid and acyl sugar production in *Solanum pennellii* and *Nicotiana benthamiana*. Plant Physiol 148(4): 1830–1846 18931142 10.1104/pp.108.129510 PMC2593661

